# Hippocampal neurons isolated from rats subjected to the valproic acid model mimic in vivo synaptic pattern: evidence of neuronal priming during early development in autism spectrum disorders

**DOI:** 10.1186/s13229-021-00428-8

**Published:** 2021-03-06

**Authors:** Marianela Evelyn Traetta, Martín Gabriel Codagnone, Nonthué Alejandra Uccelli, Alberto Javier Ramos, Sandra Zárate, Analía Reinés

**Affiliations:** 1 Instituto de Biología Celular y Neurociencia “Prof. E. De Robertis” (IBCN), Facultad de Medicina, CONICET - Universidad de Buenos Aires, Calle Paraguay 2155 3er piso, 1121 Ciudad de Buenos Aires, Argentina; 2 Cátedra de Farmacología, Facultad de Farmacia y Bioquímica, Universidad de Buenos Aires, Buenos Aires, Argentina; 3 Departamento de Histología, Embriología, Biología Celular y Genética, Facultad de Medicina, Universidad de Buenos Aires, Buenos Aires, Argentina; 4 Instituto de Investigaciones Biomédicas (INBIOMED), CONICET - Universidad de Buenos Aires, Buenos Aires, Argentina

**Keywords:** Autism spectrum disorders, VPA model, Synapse, NCAM, Hippocampus, Adhesion molecules

## Abstract

**Background:**

Autism spectrum disorders (ASD) are synaptopathies characterized by area-specific synaptic alterations and neuroinflammation. Structural and adhesive features of hippocampal synapses have been described in the valproic acid (VPA) model. However, neuronal and microglial contribution to hippocampal synaptic pattern and its time-course of appearance is still unknown.

**Methods:**

Male pups born from pregnant rats injected at embryonic day 10.5 with VPA (450 mg/kg, i.p.) or saline (control) were used. Maturation, exploratory activity and social interaction were assessed as autistic-like traits. Synaptic, cell adhesion and microglial markers were evaluated in the CA3 hippocampal region at postnatal day (PND) 3 and 35. Primary cultures of hippocampal neurons from control and VPA animals were used to study synaptic features and glutamate-induced structural remodeling. Basal and stimuli-mediated reactivity was assessed on microglia primary cultures isolated from control and VPA animals.

**Results:**

At PND3, before VPA behavioral deficits were evident, synaptophysin immunoreactivity and the balance between the neuronal cell adhesion molecule (NCAM) and its polysialylated form (PSA-NCAM) were preserved in the hippocampus of VPA animals along with the absence of microgliosis. At PND35, concomitantly with the establishment of behavioral deficits, the hippocampus of VPA rats showed fewer excitatory synapses and increased NCAM/PSA-NCAM balance without microgliosis. Hippocampal neurons from VPA animals in culture exhibited a preserved synaptic puncta number at the beginning of the synaptogenic period in vitro but showed fewer excitatory synapses as well as increased NCAM/PSA-NCAM balance and resistance to glutamate-induced structural synaptic remodeling after active synaptogenesis. Microglial cells isolated from VPA animals and cultured in the absence of neurons showed similar basal and stimuli-induced reactivity to the control group. Results indicate that in the absence of glia, hippocampal neurons from VPA animals mirrored the in vivo synaptic pattern and suggest that while neurons are primed during the prenatal period, hippocampal microglia are not intrinsically altered.

**Conclusions:**

Our study suggests microglial role is not determinant for developing neuronal alterations or counteracting neuronal outcome in the hippocampus and highlights the crucial role of hippocampal neurons and structural plasticity in the establishment of the synaptic alterations in the VPA rat model.

## Background

Autism spectrum disorders (ASD) are a group of developmental disabilities with an early onset characterized by varying degrees of impairment in social interaction, verbal and nonverbal communication and repetitive and stereotyped behavior [1]. Mounting evidence affirms that both genetic and environmental factors are deeply involved in the etiopathogenesis of these disorders [2–5].

Different non-exclusive hypotheses have been stated to shed light on autism physiopathology. It has been reported that ASD patients show altered brain connectivity described as hyper-functioning of local neural microcircuits concomitantly with long-range hypoconnectivity [6, 7]. Other studies highlight the importance of a disruption in the excitatory/inhibitory balance in different brain areas [8, 9]. Besides, strong genetic evidence concerning alterations in adhesion molecules and other synaptic proteins has defined ASD as developmental synaptopathies [10–12]. In particular, polymorphisms in the neural cell adhesion molecule (NCAM) and its polysialylation enzyme (ST8SiaII) genes have been associated with higher risk for ASD [2, 13]. Moreover, levels of NCAM have been found altered in ASD patients [14–16]. This molecule, mainly localized in excitatory synapses, plays an adhesive role and participates in vesicular recruitment and synapse efficacy [17], while its polysialylated form (PSA-NCAM) is involved in synaptogenesis, dendritic outgrowth and synapse plasticity [18, 19]. In fact, PSA-NCAM has been shown to negatively modulate N-methyl-D-aspartate (NMDA) receptor [20, 21]. It is interesting to note that a tight regulation of NCAM/PSA-NCAM balance has proved to be critical for the proper development and function of the central nervous system [22, 23].

Considering the key role glial cells play in brain function [24, 25], it is not surprising that another hypothesis points out the neuroinflammation found in ASD patients as a major contributor in the etiopathology of these disorders [26, 27]. Microglia are particularly important during brain development due to its participation in synapse formation and pruning [28–30]. Indeed, it has been proposed that alterations in synaptic pruning may lead to developmental disorders [31, 32]. It is worth mentioning that an early microglia depletion negatively impacts on social behavior [33, 34]. Also, altered synaptic and dendritic profiles may also trigger an exacerbated microglia inflammatory response [35]. Therefore, identifying the nature of microglia changes as intrinsic or neuronal-induced may lead to a better understanding of the contribution of these cells in pathologies involving neuroinflammation. Whether neurons, microglia or both cell types contribute to synaptic alterations in ASD is a matter of present investigation.

In the preclinical field, prenatal exposure to valproic acid (VPA) is a well-validated ASD animal model since it mimics the main behavioral and neuroanatomical alterations found in ASD patients [36–38]. This experimental model of idiopathic ASD shows both neuronal and glial changes in different brain regions [38–40]. The prefrontal cortex, an area involved in sociability and emotional processing, has been extensively studied in VPA animals and results match those described in ASD patients. For instance, enhanced connectivity and plasticity and increased structural synaptic markers [i.e., synaptophysin (SYN)] have been reported in VPA animals [38, 41, 42]. On the contrary, the hippocampus, a key structure of the limbic system implicated in exploration and social behavior [43, 44], remains scarcely explored in VPA animals and also poorly understood in ASD [45–48]. Remarkably, a decrease in the dendritic tree of hippocampal neurons has been shown both in VPA animals and ASD patients [49, 50]. Moreover, we previously reported a particular synaptic profile in the hippocampus of VPA animals characterized by an altered SYN pattern and NCAM/PSA-NCAM imbalance [38]. Neuroinflammation is also a common trait found in different brain regions of VPA animals [38, 39]. Even though microglial alterations seem to be age- and area-specific [39], the role of these glial cells in the hippocampus remains controversial. Overall, the VPA model raises as a useful tool to evaluate neuronal and glial participation in the development of the distinctive synaptic pattern in ASD.

The aim of the present study was to explore the neuronal contribution to synaptic changes described in ASD. As microglia play roles in synapse development and pruning, we also considered this cell type in our study. Considering the critical role of the hippocampus in the core symptoms of ASD and the scarce and intriguing results reported in this brain region, we focused on hippocampal synapses and microglia by using the rat VPA model. To shed light on neuronal and microglia contribution to hippocampal synaptic pattern, particularly that related to the behavioral deficits described in VPA rats, we used an in vivo experimental design to evaluate when synapse and microglia changes were established. We also developed an in vitro approach to study the neuronal and microglia component individually. Our work shows that hippocampal synaptic changes are established postnatally after the neonatal period in close association with VPA behavioral deficits. While purified microglia cells are not primarily affected, isolated hippocampal neurons from VPA animals mimic the in vivo synaptic pattern and proved resistant to structural remodeling. Thus, we provide evidence that hippocampal neurons are primed during the prenatal period. Therefore, our study highlights the crucial role of hippocampal neurons and their structural plasticity in the establishment of the synaptic alterations observed in the VPA model.

## Materials and methods

### Animals and drugs

Wistar rats (Facultad de Ciencias Exactas y Naturales, UBA) were housed in an air-conditioned room (20 ± 2 °C) and maintained on a 12-h light/dark cycle with food and water ad libitum. Experiments were carried out in accordance with the Guide for the Care and Use of Laboratory Animals provided by the NIH, USA. The experimental protocols were approved by the Ethics Committee for the Care and Use of Laboratory Animals of the School of Pharmacy and Biochemistry at the University of Buenos Aires (Approval No. 180613-1 and 2320). Special care was taken to minimize the number of animals used and their suffering.

All chemicals used were of analytical grade. Sodium valproate from Parafarm (Droguería Saporiti S.A.C.I.F.I.A.) was used to establish the VPA model. All reagents used in culture were purchased from Thermo Fisher Scientific Inc. Dizocilpine (5S,10R-(+)-5-Methyl-10,11-dihydro-5H-dibenzo[a,d]cyclohepten-5,10-imine maleate), also known as MK-801, 6-cyano-7-nitroquinoxaline-2,3-dione (CNQX) and lipopolysaccharide (LPS, *E. Coli* O26:B6—L3755) were purchased from Sigma-Aldrich Co. FM4-64 dye was obtained from Fisher Scientific International, Inc. The following mouse monoclonal antibodies were used for immunofluorescence: anti-NCAM and anti-PSA-NCAM (DSHB Cat# 5b8, RRID: AB_528393 and DSHB Cat# 5A5, RRID: AB_528392, respectively), anti-synaptophysin (SYN; Millipore Cat# MAB329, RRID: AB_94786), anti-microtubule-associated protein 2 (MAP-2; Sigma-Aldrich Cat# M4403, RRID: AB_477193), anti-PSD-95 (Thermo Fisher Scientific Cat# MA1-045, RRID: AB_325399) and anti-GAD-67 (Millipore Cat# MAB5406, RRID: AB_2278725). Rabbit polyclonal antibodies used were anti-actin (Sigma-Aldrich Cat# A2066, RRID: AB_476693), anti-NMDA1 (NR1; Sigma-Aldrich Cat# G8913, RRID: AB_259978) and anti-Iba1 (Wako Cat# 019-19741, RRID: AB_839504); and guinea pig polyclonal anti-vGLUT1 (Millipore Cat# AB5905, RRID: AB_2301751). For nucleus staining, 4′,6-diamidino-2-phenylindoledihydrochloride (DAPI) was used (Sigma-Aldrich Co.). Anti-SYN and anti-NCAM were also used for immunoblotting. For immunofluorescence and immunoblot studies, fluorochrome- and horseradish peroxidase (HRP)-conjugated secondary antibodies were used, respectively (Jackson ImmunoResearch Laboratories, Inc).

### VPA model

**Fig. 1:**
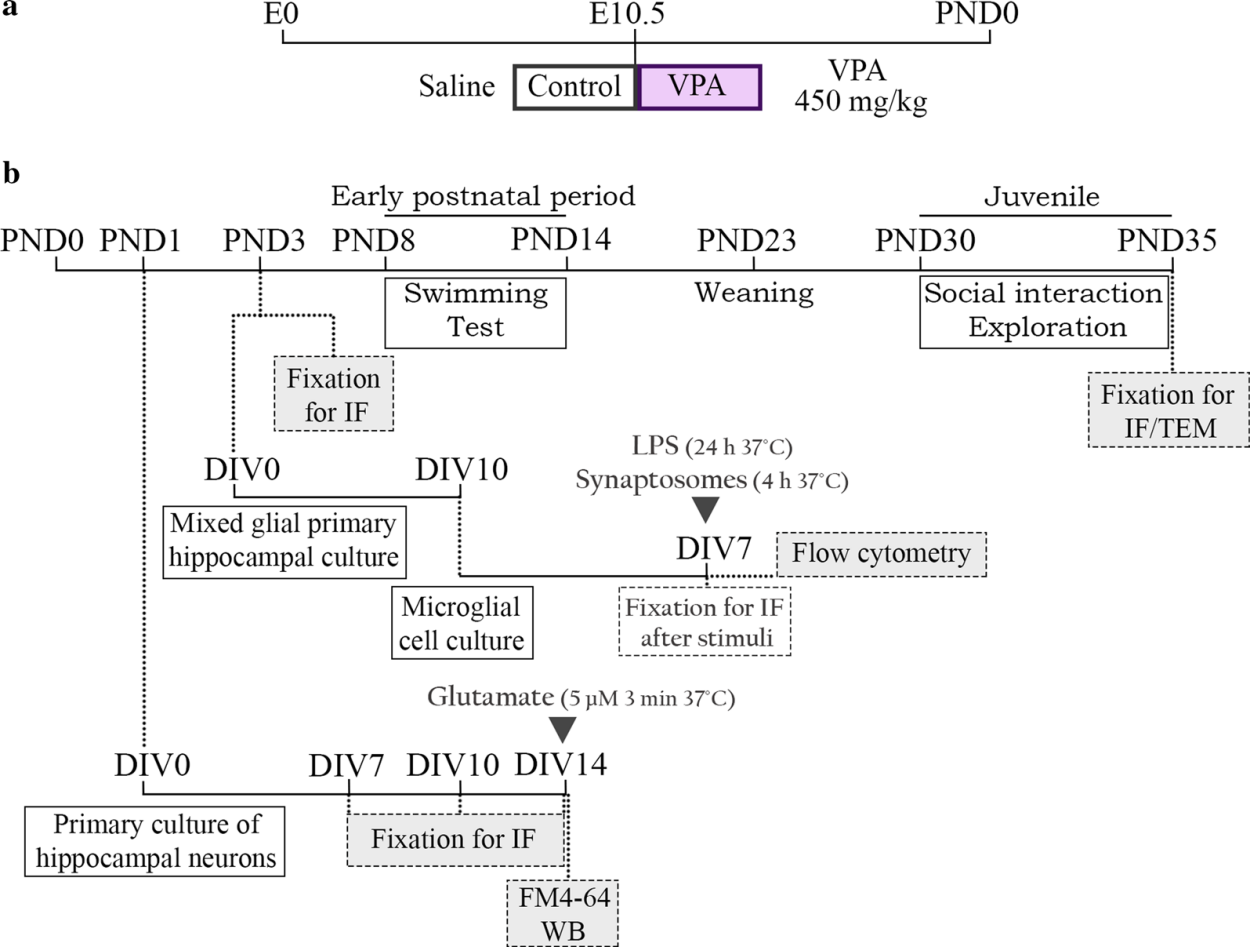
Experimental design. **a** Prenatal VPA injection defined the experimental groups. At E10.5, either VPA (450 mg/kg, i.p) or saline were intraperitoneally administrated to pregnant rats, and the corresponding pups were assigned to VPA or control groups, respectively. Postnatal day (PND) 0 was designated the day pups were born. **b** Early postnatal and juvenile VPA behavioral deficits were determined by the evaluation of the swimming performance (PND8-14) and exploration and social interaction in control and VPA animals (PND30-35). At PND3 and PND35, brains from control and VPA rats were processed for immunofluorescence (IF) assays or transmission electron microscopy (TEM) studies. The in vitro studies involved the dissection of the hippocampus from control and VPA animals to establish primary neuronal cultures (PND1) and for mixed glial primary cultures (PND3). Hippocampal neurons were fixed for immunofluorescence (IF) assays at day in vitro (DIV) 7, 10 and 14. Also, at DIV14, the FM4-64 assay was performed, and neurons were obtained for western blot (WB). Besides, a brief stimulus with glutamate was used to study structural synaptic remodeling at DIV14. In the case of glial cultures, microglial cells were obtained from mixed glial cultures. After 7 DIV, cell size and internal complexity were evaluated by flow cytometry and their morphological response to different stimuli [LPS and synaptosomes (ST)] was analyze by IF assays

VPA injection during pregnancy was performed as previously described [36, 38] with modifications. Embryonic day (E) 0 was considered the day when spermatozoa were found in vaginal smears. On E10.5, dams received a single intraperitoneal injection of saline solution or VPA (450 mg/kg, i.p.) (Fig. 1a). Sodium valproate was dissolved in saline solution (250 mg/ml). Each female was housed individually from E18 to raise their own litter until weaning at postnatal day (PND) 23. Only male pups were used for in vivo and in vitro experiments, those prenatally exposed to VPA were named VPA animals and those exposed to saline, control animals. At PND1, 3 control and 3 VPA animals were used to perform each primary culture of hippocampal neurons (Fig. 1b). Besides, starting from different litters, 4 control and 4 VPA animals were used at PND3 to obtain each mixed glial primary hippocampal culture (Fig. 1b). Pups used for neuronal and glial cultures were born from different dams and all their male siblings were used to validate the behavioral phenotype and perform biochemical analysis at PND3 and 35 (Fig. 1b). Litter size was kept at 8–12 pups (evenly distributed between male and female) per dam by culling at PND3 if necessary [38]. At PND23, males were separately housed in groups of four. The reabsorption rate and model efficacy were according to that previously described [38].

### Behavioral validation and in vivo studies in the VPA model

#### Evaluation of postnatal development and juvenile behavior

Behavioral tests were performed at early postnatal period and juvenile stage (Fig. 1b) as previously described [36] with modifications [38]. Experiments were carried out during the light phase (from 11 a.m. to 4 p.m.) employing 6–8 male pups from 4 different dams per experimental group (control or VPA).

Postnatal growth was assessed by measuring weight gain at PND7, 14, 23 and 35. At PND8, 10, 12, and 14, swimming performance was evaluated according to the position of the nose and head on the surface of the water. The swimming score was rated as follows: 0 = head and nose below the surface; 1 = nose below the surface; 2 = nose and top of head at or above the surface but ears still below the surface; 3 = the same as 2 except that the water line was at mid-ear level, and 4 = the same as 3 except that the water line was at the bottom of the ears.

At the juvenile period (PND30-35), exploratory activity was assessed in a small open field with holes in the walls. The number of hole-poking (when an animal puts its nose inside the hole) was measured during a 3 min session. At the same developmental stage, social play behavior was assessed in an acrylic plastic circular cage as previously described [38]. After 7-h isolation, 2 animals from the same group but different litters and cages were tested for 15 min. Pairs were tested in a randomized order for groups and the animals did not differ by more than 15 g in body weight. The test was recorded with a SONY CCD-TRV75 camera connected to a computer with AVerTV A833 video capture. Number of pinning (when one of the animals is lying with its dorsal surface on the floor of the test cage while the other animal is standing over him) was measured for each pair of control or VPA animals. As one pinning corresponds to both animals, sample size corresponds to the number of videos (*n* = 3 videos per group).

#### Immunofluorescence in tissue sections

In accordance with in vivo hippocampal synaptogenesis [51, 52], animals were evaluated soon after birth at PND3 (at the beginning of the synaptogenic period) and PND35 (after the synaptogenic and pruning peaks). Animal fixation and immunofluorescence technique were performed as previously described [38, 53]. At PND3, animals were decapitated, and brains fixed by immersion in fixative solution (4% w/v paraformaldehyde in 0.1 M phosphate buffer) at 4 °C for 24 h. At PND35, animals were deeply anesthetized (125 mg/kg ketamine hydrochloride and 10 mg/kg xylazine, i.p.), transcardially perfused with heparinized saline solution and fixed with 4% w/v paraformaldehyde in 0.1 M phosphate buffer. Brains were stored at -80 °C until used. Coronal 35-μm-thick tissue sections were obtained with a Leica CM1850 cryostat. Immunofluorescence assays for hippocampi were performed on sections corresponding to plates 29–35 (from bregma – 2.80 to – 4.30) of the atlas of Paxinos and Watson [54]. After permeabilization and blockage [38], anti-SYN (1:3000), anti-PSA-NCAM (1:1000), anti-NCAM (1:1000) and anti-Iba1 (1:4000) primary antibodies were used followed by fluorescent secondary antibodies. Results are expressed as mean values (± SD) of 4–6 control or VPA animals at PND3 and 4–6 control or VPA animals at PND35 (in both cases from 3 different saline and 3 VPA injected dams). Unless otherwise stated, each immunofluorescence assay consisted of 2 hippocampal serial sections of each animal per group.

#### Electron microscopy procedure and analysis

Animal fixation and sample preparation was performed as previously described [53]. At PND35, animals were deeply anesthetized as detailed above, transcardially fixed with 4% w/v paraformaldehyde and 2.5% v/v glutaraldehyde in 0.1 M phosphate buffer. Sections were post-fixed in 0.05% w/v osmium tetroxide, dehydrated and embedded in Durcupan. Ultrathin slices were stained with toluidine blue to specifically obtain the hippocampal CA3 subregion. The selected sections were contrasted with uranyl acetate and lead citrate [55] and then observed and photographed with a Zeiss 109 electron microscope equipped with a Gatan W10000 digital camera. Images from the *stratum radiatum* from 3 control and 3 VPA animals were analyzed. Synapse number was assessed by ImageJ (NIH) cell counter plugin and expressed as synapse number per µm^2^. Throughout 300 µm^2^ of the CA3 region, the average of axospinous synapses counted in each animal from the control and VPA group was 100 and 70, respectively. All synapses counted were asymmetric, defined as a pre-synapse with visible pre-synaptic vesicles and a prominent post-synaptic density consistent with an excitatory synapse morphology [56]. In addition to these features, dendritic spines were specially addressed to assure an excitatory synapse count.

### In vitro studies

#### Primary neuronal culture

Hippocampal neuronal cultures were prepared from PND1 control and VPA animals (Fig. 1b) as previously described [53]. For each culture, 3 control and 3 VPA male pups were used and male littermates were subjected to behavioral analyses to verify animal phenotype as described above. Cells were plated at a density of 5 × 10^4^ cells/cm^2^ on poly-D-lysine-coated plastic dishes for western blot or at a density of 2 × 10^4^ cells/cm^2^ on poly-D-lysine-coated glass coverslips for immunofluorescence assays. In both cases they were maintained for up to 14 days in vitro (DIV) in Neurobasal medium supplemented with 2% v/v B27 and 0.5 mM glutamine.

#### Functional labeling of pre-synaptic boutons

Loading and unloading studies were performed as previously described [53]. Hippocampal cultures (DIV14) were incubated with 15 µM FM4-64 in high potassium solution [119 mM NaCl, 45 mM KCl, 2 mM CaCl_2_, 2 mM MgCl_2_, 5.6 mM glucose, 25 mM HEPES (4-(2-hydroxyethyl)piperazine-1-ethanesulfonic acid)] for 90 s at room temperature (RT), washed, fixed and imaged. FM4-64 unloading study was carried out by performing a loading step with FM4-64 followed by a new 90 s high potassium stimulus; after washing, images were acquired every 5 s.

#### Glutamate treatment

Glutamate exposure was performed as previously described [57]. Briefly, hippocampal neurons in culture (DIV13) were exposed to 5 µM glutamate for 3 min at 37 °C. Controls were treated with PBS. Cells were fixed 20 h later (DIV14). In the experiments with glutamate receptor antagonists, neurons were pre-incubated with 10 µM MK-801 or 20 µM CNQX for 15 or 30 min, respectively. The experiments were repeated in 3 independent cultures.

#### Western blot

Cultured hippocampal neurons (DIV14) from control and VPA animals were solubilized in RIPA buffer [10 mM Tris–Cl, pH 8.0, 150 mM NaCl, 1% w/v IGEPAL (octylphenoxy poly(ethyleneoxy)ethanol), 0.5% w/v sodium deoxycholate, 0.1% w/v sodium dodecyl sulphate] as previously described [53]. The detergent-soluble fraction was analyzed by SDS-PAGE and immunoblotting using anti-SYN (1:2000) and anti-NCAM (1:1000) primary antibodies followed by HRP-conjugated antibodies and actin as loading control. Protein concentration was determined by the Bradford protein assay and 25 µg of protein was used in each lane. Lysates were obtained from 3 independent cultures.

#### Primary microglial culture

Microglial enriched cultures were obtained from mixed glial primary cultures, which were prepared from 4 control and 4 VPA animals at PND3 (male littermates were subjected to behavioral analyses to verify animal phenotype as described above) (Fig. 1b). Mixed glial primary cultures were performed as previously described [58]. Once cells reached confluence, microglia cells were detached and re-seeded on poly-D-lysine coated glass coverslips at a density of 4 × 10^4^ cells/cm^2^ for treatments and immunofluorescence assays or at a density of 5 × 10^4^ cells/cm^2^ on plastic dishes for flow cytometry. Cultures obtained with this procedure showed > 98% Iba1 (+) microglia when compared to number of nucleus stained with DAPI.

#### Treatments on microglial cultures

##### LPS treatment

At DIV6, microglial cultures from control and VPA animals were treated with 20 ng/ml of LPS for 24 h. These conditions were selected to induce microglia reactivity without affecting cell viability in culture [59].

##### Synaptic terminals treatment

Synaptosomes obtained from naïve adult male Wistar rats were prepared as described by Phillips et al*.* [60]. At DIV7, synaptic terminals were added to microglial cultures from control and VPA animals for 4 h at 37 °C.

#### Flow cytometry and analysis

Flow cytometry was performed as previously described [61]. At DIV7, cultured microglial cells were harvested, fixed with 4% w/v paraformaldehyde and run on a Partec PAS III flow cytometer (Partec, GmbH, Münster, Germany). Data were analyzed with WinMDI 98 software. A representative region was selected on a dot plot using the control group, afterwards the same region was used to study the VPA group. The mean value of the Forward Scatter ​​(FSC) was used to determine the relative cell size and the Side Scatter mean value (SSC) was used to define internal complexity. Internal complexity (i.e. granularity) is indicative of microglia reactivity, particularly an increase in internal complexity reveals a higher phagocytic activity [62]. Microglial cells from two independent cultures were evaluated and measurements from each experimental group were run in triplicate. A representative experiment is shown.

##### Immunofluorescence on coverslips

Hippocampal neurons were fixed at DIV7, 10 and 14 and hippocampal microglia were fixed at DIV7 after treatment. Fixation time points for hippocampal neurons were selected to study arborization and synapse formation throughout synaptogenesis in vitro [63, 64]. These time points were chosen to correlate hippocampal neuronal differentiation with development [51, 52]: DIV7 corresponds to the beginning of the synaptogenic period in vitro and DIV14 corresponds to mature synapses after active synaptogenesis, which correlates to PND35 since even neuronal gene expression program in vitro and in vivo is highly conserved, in vitro it is quicker accomplished [65]. Cells on coverslips were processed for immunofluorescence as previously described [53, 57]. Primary antibodies anti-actin (1:100), anti-MAP-2 (1:500), anti-SYN (1:2000), anti-PSD-95 (1:200), anti-NR1 (1:300); anti-vGLUT1 (1:5000), anti-GAD-67 (1:1000), anti-PSA-NCAM (1:100) and anti-Iba1 (1:1500) followed by secondary antibodies and DAPI (0.5 µg/ml) were used. Assays were repeated 2–3 times employing independent cultures.

### Image acquisition and analysis

Immunofluorescence images from tissue slices and fixed cultures were captured by an epifluorescence Olympus IX81 microscope (40× magnification objective) equipped with a CCD model DP71 digital camera (Olympus). GAD-67 and phase contrast images were obtained with an Olympus IX83 microscope (40× magnification objective) equipped with a CMOS model C13440-22CU ORCA Flash 4.0 V2 digital camera from Hamamatsu Photonics. Images were analyzed using ImageJ (NIH) software.

In tissue sections, unless otherwise stated, two adjacent non-overlapping images were taken to sample the CA3 hippocampal region from each hemisphere. SYN, NCAM and PSA-NCAM immunolabeling was measured as relative immunoreactive area, quantified as positive immunoreactive area normalized to the total area corresponding to the CA3 in the field of view. Using the ImageJ (NIH) software as previously described by Codagnone et al. [38], images were transformed into gray scale and the positive immunoreactive area was defined as that which exceeded the established gray value threshold selected to differentiate the immunolabelled structures from the background. Once established for each marker, threshold values were kept constant between experimental groups. To analyze Iba1 immunolabeling, three parameters were studied: relative immunoreactive area to total area corresponding to CA3, Iba1 (+) cell number and microglia morphology by classifying Iba1 (+) cells into ramified and unramified categories. Ramified microglia were considered when a cell presented a small soma and more than two long and thin processes, while unramified showed a larger soma and few short and thick processes. Iba1 (+) cell number was estimated based on the optical fractionator method described by West et al. [66] and the area of the CA3 was calculated according to Sosa-Díaz et al. [67]. Every 20th section (665 µm apart) corresponding to plates 29–35 (from bregma – 2.80 to – 4.30) of the atlas of Paxinos and Watson [54] was processed. To measure the CA3 area in each of the three serial sections employed, both hemi-hippocampi were photographed at low magnification (10× objective, NA 0.3) and the CA3 region delimited according to the rat atlas of Paxinos and Watson [54] using the ImageJ (NIH) software. The CA3 areas measured in the serial sections were averaged for total area estimation per animal. Cells were counted manually using a 40× magnification objective (NA 0.6) whose *xy* movements corresponded to two adjacent non-overlapping fields of view (equivalent to 276 × 270 × 10 µm^3^ counting frame and a 552 × 540 µm^2^ *xy* step) along the CA3 subfield. Counting spots in each section corresponded to 50% of the CA3 area. Only Iba1 (+) cells that came into focus within the optical plane were included in the measurement. The volume of the CA3 sampled region was calculated for each animal as mean CA3 area x brain thickness sampled with three serial sections. Finally, Iba1 (+) cell number/CA3 volume ratio was calculated for each animal and averaged per group.

In neuronal culture, for synaptic immunostaining patterns (SYN, PSD-95, NR1, vGLUT1, GAD-67), a single threshold was set for every condition to capture puncta. The cluster of synaptic proteins labeled with antibodies are termed synaptic puncta [68]. For each marker, a single threshold was established to clearly distinguish individual puncta from the background and to minimize the probability of including merged structures in the quantification. The size range selected to define SYN, PSD-95, NR1 and vGLUT1 puncta was 0.15–0.6 µm^2^; for GABAergic marker was 0.4–1.2 µm^2^ [53, 57, 69, 70]. Synaptic puncta per neuron was quantified as total puncta number, which relates to the number of synapses, individual puncta area as a size parameter, and total area occupied by puncta, which can be influenced by the two former parameters. Actin and PSA-NCAM were measured as total immunoreactive area per neuron. Dendritic tree was determined by subtracting the area of the soma to the total MAP-2 area. Synapses relative to dendritic tree were quantified as SYN puncta number relative to 30 µm length of secondary dendrites labeled with MAP-2 to standardize the procedure across experimental conditions. FM4-64 loading analysis was quantified as number, size, and total area of synaptic puncta. Unloading analysis consisted in measuring the drop in fluorescence intensity of 20 individual puncta from a unique neuron (3 neurons from each group were studied).

Microglia morphology in culture was assessed by measuring mean circularity per field using ImageJ (NIH) software. The circularity, calculated by the software as 4*π* (area/perimeter^2^), can range from 1 to 0, being 1 a perfectly circular cell and 0 an elongated or ramified microglia. This parameter is an index of microglia reactivity, a more circular shape is associated to a more reactive cell in vitro [59, 71]*.* Besides, cell area was calculated as the mean Iba1 (+) cell area per field, by setting a threshold using ImageJ (NIH) software.

Final figures were created with Photoshop CS6. In case of bright and contrast adjustments, they were equally applied to all groups.

### Statistical analysis

InfoStat (Facultad de Ciencias Agropecuarias, Universidad Nacional de Córdoba, Argentina) software was used to perform the statistical analysis. When comparing two normal, independent variables, a Student’s *t* test was applied; otherwise, the analysis was performed using a non-parametric Mann–Whitney *U* test. In the case of comparing more than two normal variables, once variance homogeneity was proved, two-way or one-way analysis of variance (ANOVA) followed by Tukey test was used, if not, a non-parametric Kruskal Wallis test was performed. Each analysis and level of significance between groups is described in the figure legend. All (*) represent a parametric test, while (#) represents a non-parametric one. Statistical significance was set at *p* < 0.05.

## Results

### Synapse number reduction in the hippocampus of VPA rats was established postnatally after the neonatal period

**Fig. 2:**
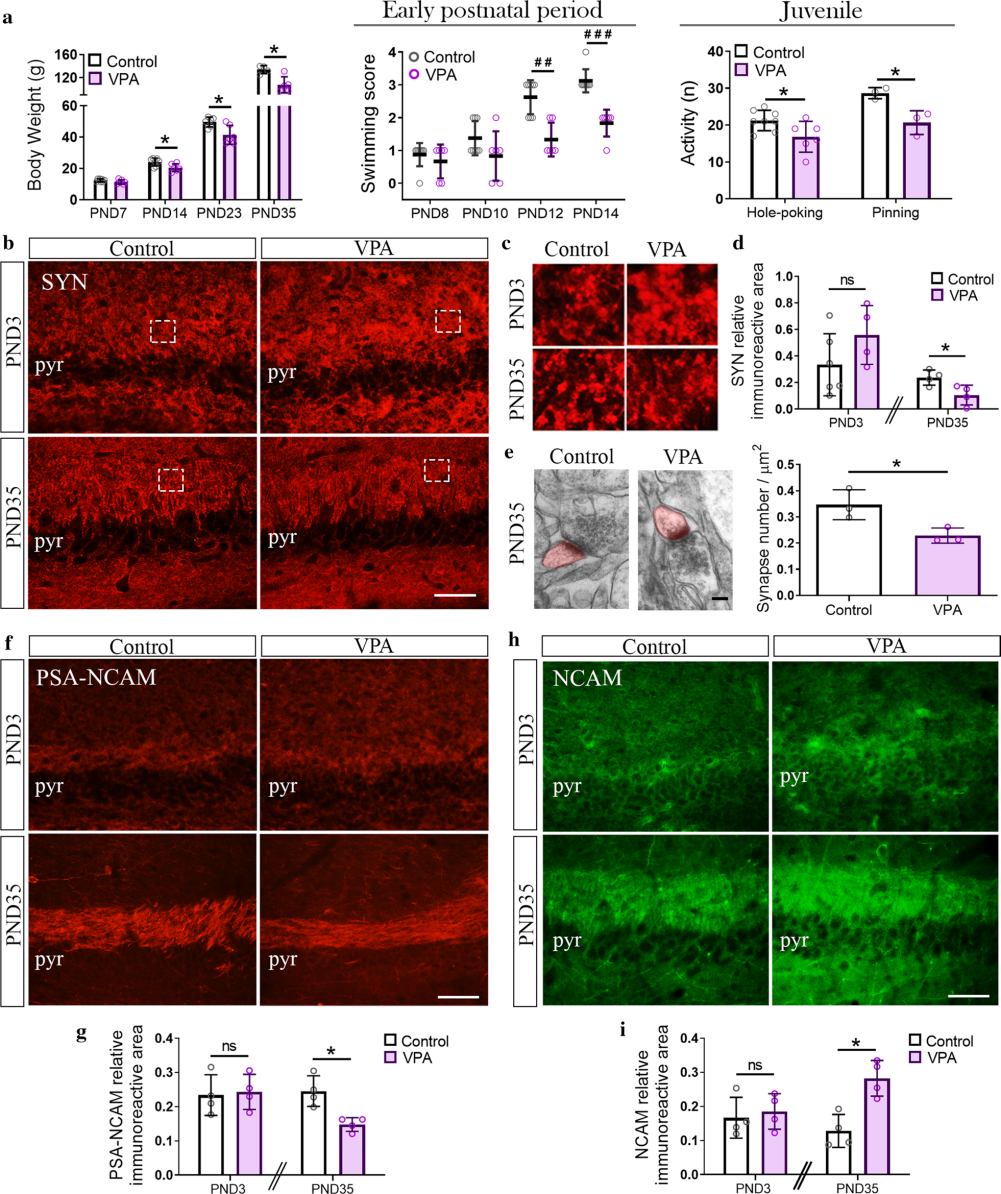
Hippocampal synapse reduction and increased NCAM/PSA-NCAM balance in VPA animals were established after the neonatal period. **a** VPA animals exhibited lower body weight at PND14, 23 and 35, worse swimming performance than controls at PND12 and 14 and reduced number of hole-poking and pinning at PND30-35. **b** Representative photomicrographs of CA3 hippocampal region from control and VPA animals immunostained for synaptophysin (SYN) at PND3 and 35. **c** Insets (4 × magnification) detail SYN immunostaining pattern. **d** SYN relative immunoreactive area quantification showed the absence of statistical differences at PND3 but a robust reduction at PND35. **e** Quantification of excitatory synapses (asymmetric) in the CA3 hippocampal region by electron microscopy depicted fewer synapses in VPA animals at PND35. **f** CA3 hippocampal region from control and VPA animals immunostained for PSA-NCAM at PND3 and 35. **g** VPA animals displayed conserved and reduced PSA-NCAM levels at PND3 and PND35, respectively. **h** CA3 hippocampal region from control and VPA animals immunostained for NCAM at PND3 and 35. **i** Quantification of NCAM relative immunoreactive area confirmed the absence of statistical differences at PND3 and an increase at PND35 in the VPA group. Results are expressed as mean values (± SD; **a**: control *n* = 6–8 animals, VPA *n* = 6–8 animals; *n* = 3 videos per group in the case of pinning assessment; **d**: control *n* = 4–6 animals, VPA *n* = 4 animals; **e**: control n = 3 animals, VPA n = 3 animals; **g**, **i:** control *n* = 4 animals, VPA *n* = 4 animals). ns: non-significant; *p < 0.05 between bars by Student’s *t* test; ^##^*p* < 0.01; ^###^*p* < 0.001 between groups by Mann–Whitney *U* Test. Scale bars: 50 µm (**b**, **f**, **h**); 100 nm (**e**). pyr: soma of CA3 pyramidal neurons

Alterations in synapse markers have been previously described in the hippocampus of VPA animals at postnatal ages concomitant with exploratory and social deficits [38, 72, 73]. In fact, the structural synaptic marker synaptophysin (SYN) is reduced in the CA3 hippocampal region of VPA animals in the juvenile period [38]. To address when this synapse change is established, we evaluated whether SYN labeling diminution was recognized soon after birth in the neonatal period or later during the juvenile postnatal period. To this aim, we first addressed early postnatal maturation and, in the juvenile period, autistic-like behavior in each animal. At early postnatal period, VPA rats showed significantly lower body weight at PND14 and scored lower than controls in the swimming test at PND12; both parameters did not reach statistical significance at PND7 and 8, respectively, indicating that growth and maturation deficits in the VPA group became evident later than PND7 (Fig. 2a). Since exploratory and social deficits emerge in VPA rats at the juvenile period, exploratory activity and social interaction were evaluated at PND30-35. Juvenile VPA animals showed an exploratory deficit measured as fewer hole-pokings compared to controls and exhibited social interaction impairment shown as fewer pinnings (Fig. 2a).

Therefore, we evaluated SYN immunolabeling in the hippocampus at PND3, before early behavioral deficits were evidenced and before synaptogenic and synaptic pruning peaks had occurred [51, 74–76]. The same analysis was performed at PND35 once the VPA phenotype was fully demonstrated. The study was done in the CA3 since synapses in this subregion belong to the intrahippocampal circuit, thus excluding the contribution of non-hippocampal neurons [77]. At PND3, the CA3 of VPA animals showed similar SYN immunostaining to control rats (Fig. 2b). SYN immunolabeling displayed the typical synapse pattern depicted as protein clusters (Fig. 2c). Quantification revealed the absence of statistical differences in SYN relative immunoreactive area at PND3 (Fig. 2d). In contrast, at PND35 the CA3 hippocampal region of VPA animals showed a significant reduction in SYN immunolabeling (Fig. 2b, d). As this SYN reduction suggested a decrease in synapse number, we assessed this parameter by morphological evaluation of asymmetric synapses in the *stratum radiatum* of the CA3 by electron microscopy. This *stratum* is mostly comprised by excitatory synapses (asymmetric) from intrahippocampal pyramidal neurons [77]. VPA rats showed fewer asymmetric synapses in this hippocampal region compared to control animals at PND35 (Fig. 2e). Thus, results indicate that the reduction in the number of excitatory synapses in the hippocampus of VPA animal was established postnatally after the neonatal period, most probably after the synaptogenic and synaptic pruning peaks, and concomitantly with the development of the VPA phenotype.

### Increased NCAM/PSA-NCAM balance in the hippocampus of VPA animals was concomitant with the reduction in synapse number

We previously reported that male VPA rats exhibit an increase in NCAM along with a decrease in PSA-NCAM expression in the CA3 hippocampal region at PND35 [38]. Since NCAM participates in vesicle recruitment and synapse stability [17] and PSA-NCAM has a key role in dendritic outgrowth and synapse formation [18, 19], we evaluated whether synaptic changes in the hippocampus of VPA animals were accompanied by alterations in NCAM/PSA-NCAM balance. At PND3, PSA-NCAM and NCAM levels did not differ between control and VPA pups (Fig. 2f–i). Remarkably, at PND35 whereas PSA-NCAM decreased, NCAM increased in the CA3 hippocampal region of VPA rats (Fig. 2f–i). These results indicate that the increase in NCAM/PSA-NCAM balance was concomitant with synapse number reduction and that it was established postnatally after the neonatal period.

### After active synaptogenesis, hippocampal neurons isolated from VPA animals formed fewer synapses in vitro

**Fig. 3:**
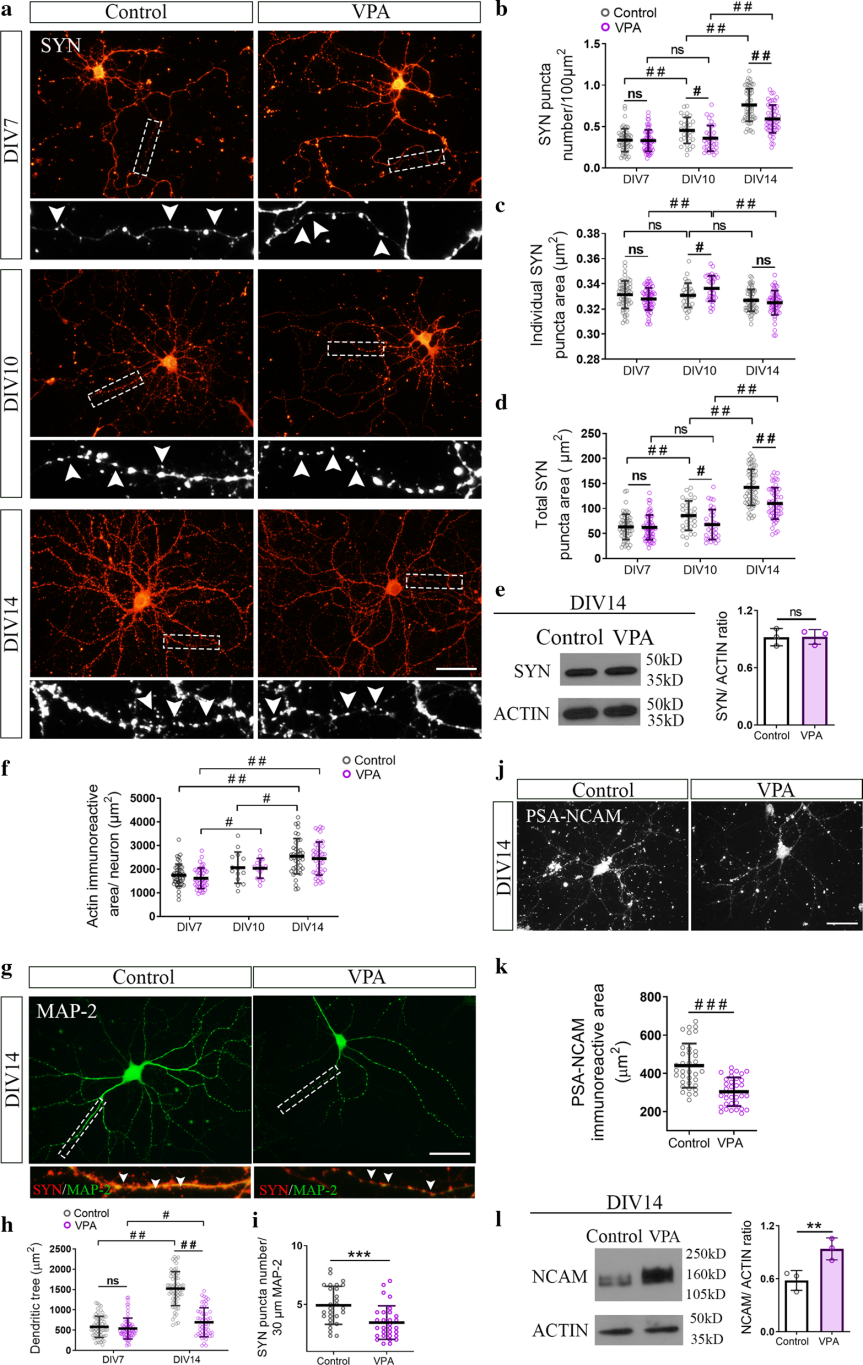
Hippocampal neurons isolated from VPA animals mimicked, in vitro*,* the synaptic pattern found in vivo. Hippocampal neurons isolated from control and VPA animals were cultured in the absence of glia. **a** Synaptophysin (SYN) immunostaining of hippocampal neurons at DIV7, 10 and 14. Insets (×4 magnification) detail SYN immunostaining pattern. **b** Neurons from VPA animals showed conserved SYN puncta number at DIV7 but reduced at DIV10 and 14 compared to controls. **c** No deep changes were observed in synaptic size throughout differentiation. **d** Quantification of total SYN puncta area matched puncta number results. **e** No statistical differences in total SYN expression by western blot, ruling out changes in SYN expression. **f** Actin arborization was similar between groups throughout neuronal differentiation. **g** MAP-2 immunostaining and SYN/MAP-2 co-staining (×3 magnification insets) at DIV14. **h** MAP-2 immunoreactive area quantification revealed a preserved dendritic tree at DIV7 but a smaller one at DIV14 in neurons from VPA animals. **i** SYN puncta number decreased regardless of MAP-2 immunostaining reduction in neurons from VPA animals at DIV14. **j, k** Decreased PSA-NCAM immunoreactivity in hippocampal neurons from VPA animals (DIV14). **l** Increased NCAM expression in hippocampal neurons from VPA animals (DIV14). Results are expressed as mean values (± SD; **b-d, h, k** control n = 30–60 neurons, VPA n = 30–60 neurons from 3 independent cultures; **e, l** control *n* = 3 lysates, VPA *n* = 3 lysates from 3 independent cultures; **f, i:** control *n* = 30–40 neurons, VPA *n* = 30–40 neurons, except for **f** DIV10 n = 15 neurons, from 2 independent cultures). **b, c, d, f** and **h** ns: non-significant; ^#^*p* < 0.05; ##p < 0.01 between groups by Kruskal Wallis; **e, i, l** ns: non-significant; **p < 0.01; ***p < 0.001 between groups by Student’s *t* test; **k** ^###^p < 0.001 between groups by Mann–Whitney *U* Test. Scale bar: 50 µm

We studied the neuronal contribution to the hippocampal synapse alterations observed in vivo by performing an in vitro assessment of neuronal differentiation in the absence of glial cells. Before the active period of synapse formation in vitro (DIV7), cultured hippocampal neurons isolated from VPA pups (PND1) showed similar SYN puncta number, individual and total puncta area when compared with neurons isolated from control animals (Fig. 3a–d). It is accepted that puncta number and individual puncta size indicate synapse number and pre-/post-synapse size, respectively [53, 57, 69, 70]. During the active period of synapse formation in vitro (DIV7-14) [64, 65], hippocampal neurons from both experimental groups increased SYN puncta number throughout differentiation (Fig. 3a, b). However, neurons from VPA animals showed fewer SYN puncta number at DIV10 and 14 when compared with neurons from control animals (Fig. 3a, b). Thus, even though neurons from control and VPA animals showed similar SYN puncta number at DIV7 and synaptogenesis progressed in both groups, neurons from VPA animals were unable to match the control group throughout the synaptogenic period in vitro (Fig. 3a, b). Quantification of individual puncta size did not reveal any robust change in synaptic size throughout differentiation (Fig. 3c). Besides, changes in total SYN puncta area confirmed the results obtained with SYN puncta number from DIV7 to DIV14 in neurons from both control and VPA animals (Fig. 3d). Thus, hippocampal neurons isolated from VPA animals showed in vitro a time-dependent reduction in SYN puncta number in the absence of glia. Also, preserved total SYN expression in hippocampal neurons from VPA animals (Fig. 3e) ruled out decreased synapse number due to a reduction in the level of this structural synaptic protein and suggests that synapse formation and/or stability could be compromised in hippocampal neurons from VPA animals.

Since synapse number is deeply influenced by neuronal arborization [78], we studied neurite differentiation in cultured neurons immunostained for actin and MAP-2. Neurons from VPA animals depicted similar actin arborization throughout differentiation when compared with neurons from control animals (Fig. 3f). However, slight differences were observed when evaluating the progression of differentiation, since neurons from control animals showed a significant actin increase between DIV10 and DIV14 while neurons from VPA animals did so between DIV7 and DIV10 (Fig. 3f). The dendritic tree was evaluated by MAP-2 immunostaining at DIV7 and DIV14 when no changes and a deep decrease in synaptic puncta were observed, respectively. While at DIV7, neurons from VPA animals showed a conserved dendritic tree, at DIV14, a significant reduction was observed compared to the control group (Fig. 3g, h). Although the latter might account for the decrease in SYN puncta number at DIV14, quantification of SYN puncta number relative to dendrite length revealed that the decrease in synapse number was independent of the reduction in the dendritic tree in neurons from VPA animals (Fig. 3i). These results also point out synapse formation and/or stability as the processes involved in the reduction of synapse number in hippocampal neurons from VPA animals. Overall, these results show that, in the absence of glia, hippocampal neurons from VPA animals formed fewer synapses after active synaptogenesis and thus, mimicked in vitro the time-dependent synaptic pattern described in vivo.

### Increased NCAM/PSA-NCAM balance in hippocampal neurons isolated from VPA animals was concomitant with the reduction in synapse number

Considering that hippocampal neurons isolated from VPA animals mimicked in vitro the time-dependent changes in synapse number previously seen in vivo*,* we tested whether NCAM/PSA-NCAM balance was affected after active synapse formation. At DIV14, cultured neurons isolated from VPA animals showed an increase in NCAM/PSA-NCAM balance concomitant with the reduction in synapse number, depicted as a decrease in PSA-NCAM immunolabeling (Fig. 3j, k) and an increase in NCAM expression (Fig. 3l). Thus, hippocampal neurons from VPA animals also mimicked in vitro the increase in NCAM/PSA-NCAM balance shown in vivo.

### Reduced excitatory synapses in hippocampal neurons isolated from VPA animals

**Fig. 4:**
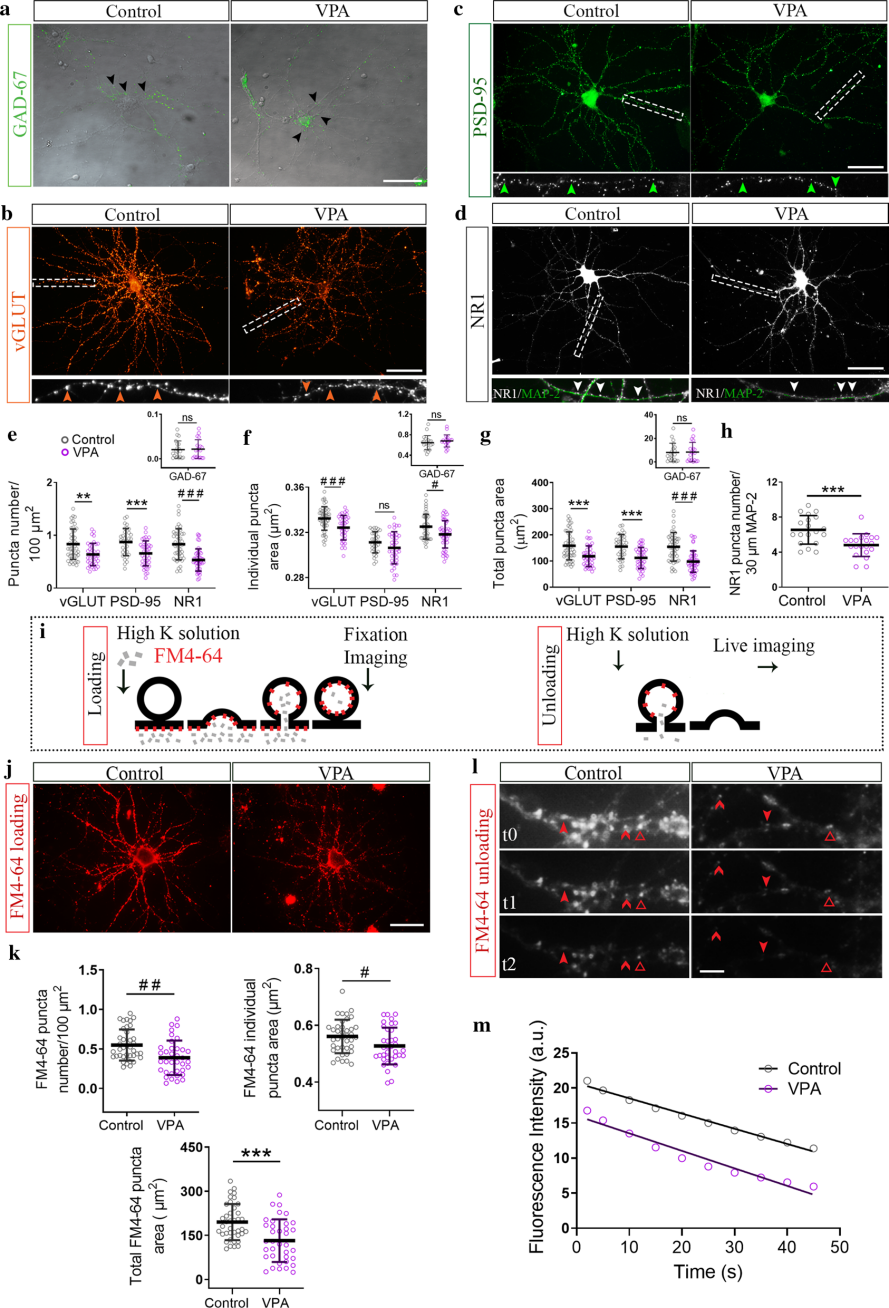
Hippocampal neurons isolated from VPA animals formed fewer glutamatergic synapses after active synaptogenesis in vitro. Hippocampal neurons isolated from control and VPA animals were cultured in the absence of glia. **a** GABAergic synapses immunostained for pre-synaptic marker GAD-67 and overlapped with phase contrast images (DIV14). Glutamatergic synapses were immunostained (DIV14) for **b** vesicular glutamate transporter vGLUT1 (3 × magnification inset depicts vGLUT1 synaptic puncta), **c** post-synaptic marker PSD-95 (3 × magnification inset shows PSD-95 synaptic puncta) and **d** NMDA receptor subunit NR1 (3 × magnification inset shows NR1/MAP2 co-staining). **e** Puncta number quantification revealed no statistical changes in GAD-67 and a reduction in vGLUT1, PSD-95 and NR1 in neurons from VPA animals. **f** vGLUT1 and NR1 individual puncta exhibited smaller size in neurons from VPA animals. **g** Total puncta area was coincident with puncta number. **h** Decreased NR1 puncta number regardless dendritic tree reduction in neurons from VPA animals. **i** Experimental protocol for FM4-64 synaptic loading and unloading. **j** FM4-64 loading images revealed a reduced number of active pre-synaptic terminals in neurons from VPA animals. **k** Analysis of FM4-64 puncta parameters confirmed fewer functional synapses, smaller functional vesicular pools and a diminished total puncta area in neurons from VPA animals. **l** Representative images at different time points showed conserved unloading capacity of neurons from VPA animals. Arrows depict fluorescent puncta over time analyzed in unloading studies; *t*0 = 5 s, *t*1 = 25 s and *t*2 = 50 s. **m** Conserved unloading kinetics in neurons from VPA animals (Control: m: − 0.2169 s^−1^ and VPA: m: − 0.2498 s^−1^, no significant differences between slopes, Student’s *t* test). Results are expressed as mean values (± SD, **e–h** control *n* = 20–50 neurons, VPA *n* = 20–50 neurons from 2 independent cultures; **k** control *n* = 39 neurons, VPA *n* = 36 neurons from 2 independent cultures). **p < 0.01; ***p < 0.001 between groups by Student’s *t* test and ns: non-significant; ^#^*p* < 0.05; ^##^*p* < 0.01; ^###^*p* < 0.001 between groups by Mann–Whitney *U* Test. Scale bars: 50 µm (**a**–**d**, **j**); 5 µm (**l**)

Considering the decrease in SYN puncta number observed in hippocampal neurons isolated from VPA animals when studied in culture at DIV14, we evaluated glutamatergic and GABAergic contribution to such synapse number reduction. GABAergic synapses were found unaltered in neurons from VPA animals immunostained for GAD-67 (Fig. 4a) and quantified as GAD-67 puncta number (Fig. 4e), individual puncta size (Fig. 4f) and total puncta area (Fig. 4g). To study glutamatergic synapses, vGLUT1, PSD-95 and NR1 immunolabeling were assessed. Neurons from VPA animals showed a decrease in number and size of glutamatergic pre-synapses evaluated by vGLUT1 immunolabeling (Fig. 4b, e–g). This result was in line with fewer PSD-95 puncta, confirming a diminution of glutamatergic synapses (Fig. 4c, e–g). Moreover, the constitutive subunit of NMDA receptor NR1 labeling exhibited not only a decreased puncta number but also smaller NMDA receptor clusters (Fig. 4d, e–g). Similar to what was described for SYN reduction, the diminution in NR1 puncta number was independent of the dendritic tree modification (Fig. 4h). These results indicate that hippocampal neurons isolated from VPA animals also mimicked the reduction in the number of excitatory synapses seen in the hippocampus of juvenile VPA animals.

### Neurons from VPA animals exhibited fewer and smaller functional synapses with preserved unloading vesicle kinetics in vitro

We performed the labeling of pre-synaptic boutons with the FM4-64 dye (Fig. 4i) to determine whether synapses formed in vitro by neurons from VPA animals were functional. Cultured neurons from VPA animals exhibited fewer and smaller functional synapses as seen by reduced number and size of FM4-64 labeled puncta (Fig. 4j, k). However, dye unloading after a depolarizing stimulus remained unaltered compared to neurons obtained from control rats (Fig. 4l, m) indicating that even though there were fewer synapses and smaller vesicular pools in neurons isolated from VPA animals, they exhibited conserved unloading vesicle kinetics.

### Hippocampal neurons isolated from VPA animals resisted structural synapse remodeling in vitro

**Fig. 5:**
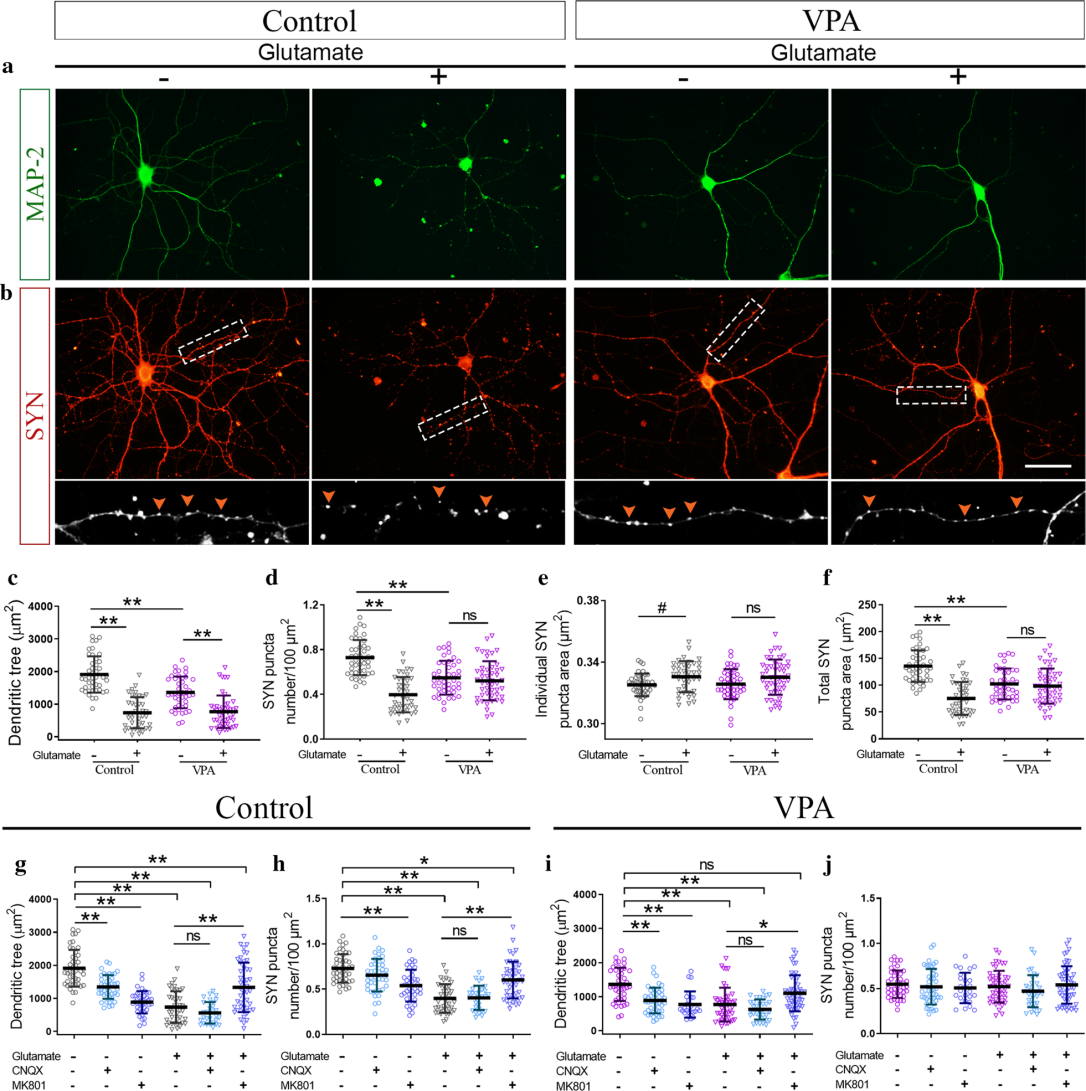
Hippocampal neurons isolated from VPA animals showed resistance to glutamate-induced synapse remodeling in vitro. Hippocampal neurons in culture isolated from control and VPA animals were exposed to glutamate (5 µM, 3 min) and immunostained (DIV14) for **a** MAP-2 and **b** SYN (×4 magnification insets show SYN puncta pattern). **c** Quantification of MAP-2 dendritic area revealed that neurons for both experimental groups remodeled their dendritic tree after glutamate exposure. **d** SYN puncta number quantification depicted a reduction in neurons from control animals in response to glutamate exposure which was absent in neurons from VPA animals. **e** Neurons from control animals showed an increase in individual SYN puncta area when exposed to glutamate, while neurons from VPA animals showed a preserved puncta size. **f** In accordance with puncta number reduction, total SYN puncta area decreased in the control group. **g, i** Pre-treatment with MK-801 prevented dendritic tree retraction in neurons from both control and VPA animals. **h, j** Pre-treatment with MK-801 prevented glutamate induced SYN puncta reduction in control neurons and showed no effect in neurons from VPA animals. Results are expressed as mean values (± SD, control *n *= 45 neurons, VPA *n* = 45 neurons, except for: **g–j** glutamate plus CNQX *n* = 30 neurons, **i, j** MK-801 *n* = 25 neurons, from 3 independent cultures). **c–f** ns: non-significant; *p < 0.05; **p < 0.01 between groups by two-way ANOVA followed by Tukey test; ns: non-significant; ^#^*p* < 0.05 between groups by Kruskal Wallis test; **g–j:** ns: non-significant; *p < 0.05; **p < 0.01 between groups by one-way ANOVA followed by Tukey test. Scale bar: 50 µm

It has been reported that a brief exposure to a low glutamate concentration induces structural remodeling characterized by dendritic retraction and synapse loss in the absence of neuronal death. PSA-NCAM and NCAM display crucial roles in this glutamate-induced synapse remodeling, which is NMDA receptor-dependent [57]. Thus, considering the increased NCAM/PSA-NCAM balance seen in hippocampal neurons from VPA animals at DIV14, this strategy emerges as an excellent tool to study synapse remodeling as a functional approach. After glutamate exposure, both neurons from control and VPA animals reduced their dendritic tree (Fig. 5a, c) but only neurons from control animals diminished their SYN puncta number and increased their individual puncta size (Fig. 5b, d–f). Furthermore, the remodeling was proved to be NMDA receptor-dependent in both experimental groups, since pre-incubation with MK-801, an NMDA antagonist, but not CNQX, an AMPA antagonist, prevented the alterations induced by glutamate in both groups (Fig. 5g–j). These results indicate that even when neurons from both VPA and control animals responded to glutamate stimulus with dendritic retraction, only neurons from control animals exhibited synaptic changes, thus suggesting that hippocampal neurons isolated from VPA animals exhibit higher resistance to glutamate-induced synapse remodeling. These results suggest that distinctive synaptic features of hippocampal synapses from VPA animals such as NCAM/PSA-NCAM balance could underlie the resistance to structural plasticity.

### In vivo and in vitro evidence of lack of microglial response to hippocampal synaptic reduction in VPA animals

**Fig. 6:**
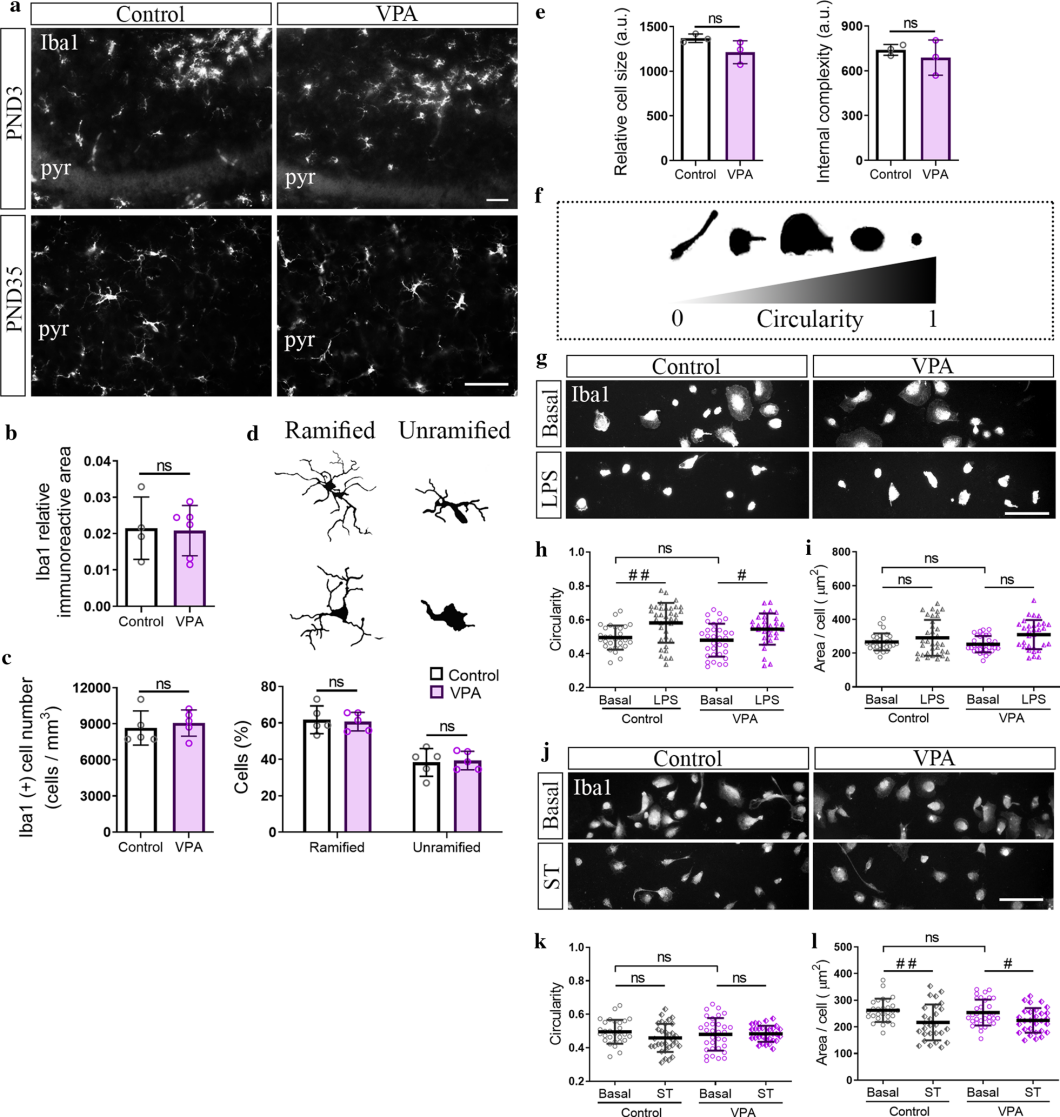
Absence of microgliosis and preserved response to stimuli of hippocampal microglia from VPA animal. **a** CA3 hippocampal region from control and VPA animals immunostained for Iba1. At PND3, qualitative analysis of microglia showed a similar immunostaining pattern in both groups. At PND35, **b** Iba1 relative immunoreactive area and **c** number of Iba1 (+) cells were conserved in the VPA group. **d** Cell schematic representation shows microglia morphology classification employed. No statistical differences were observed between groups when number of ramified and unramified cells was counted related to total number of Iba1 (+) cells. **e** Microglia isolated from VPA animals and cultured in the absence of neurons showed preserved size and internal complexity when analyzed by flow cytometry. **f** Representation of cell morphology in culture and its associated circularity, indicative of cell reactivity. **g** Hippocampal microglia cultures isolated from control and VPA animals and immunolabeled for Iba1 in basal conditions and after LPS stimulus. **h** Similar reactivity profile (circularity values) was observed in both groups in basal conditions; both groups responded to LPS by increasing its circularity **i** with no changes in average cell area. **j** Cultured hippocampal microglia isolated from control and VPA animals and immunolabeled for Iba1 in basal conditions and after exposure to hippocampal synaptosomes (ST). **k** No statistical changes in circularity were observed after exposure to ST in control and VPA groups. **l** After ST exposure, microglial cells from both experimental groups responded by decreasing the average cell area. Results are expressed as mean values (± SD; **b–d** control *n* = 4–5 animals, VPA *n* = 5–6 animals; **e** a representative experiment run by triplicate; **h–l** 30–35 photomicrographs per group from two independent cultures). **b–e:** ns: non-significant between bars by Student’s *t* test; **h–l** ns: non-significant; #p < 0.05; ##p < 0.01 between groups by Kruskal–Wallis Test. Scale bar: 50 µm. pyr: soma of CA3 pyramidal neurons

Our results show that a reduction in synapse number was established in the hippocampus after the neonatal period (PND3). Microglia are involved in synapse formation and synaptic pruning in early life [30]. These glial cells have been proposed to play a role in ASD neuropathology [79–81]. Some studies from our and other laboratories have evaluated microglia reactivity in the hippocampus of VPA animals, but results are still not conclusive [38, 39]. In order to elucidate whether hippocampal microglia may contribute to synapse number reduction, we studied morphological parameters of microgliosis in the CA3 hippocampal region of VPA animals at PND3 and 35, when changes in synapse number are absent or already proven, respectively. At PND3, qualitative analysis of Iba1 immunostaining showed similar microglia pattern, distribution, and density in the hippocampus of control and VPA animals (Fig. 6a). At PND35, both microglia positive area (Fig. 6b) and microglia cell number (Fig. 6c) did not differ between experimental groups. Ramified and amoeboid microglia profiles are associated with resting and reactive microglia, respectively [82, 83]. Evaluation of ramified and unramified microglia at PND35 showed similar proportion of each population both in control and VPA animals (Fig. 6d). Thus, results indicate the absence of microgliosis in the CA3 hippocampal region of VPA rats before and after the establishment of synapse number reduction.

To further study hippocampal microglia from VPA animals before the establishment of the reduction in synapse number, we isolated microglia from the hippocampus at PND3 and grew them in culture in the absence of neurons. This approach allowed us to evaluate intrinsic properties of microglia by ruling out adaptive changes induced by microenvironmental cues during the postnatal period [84–86]. In basal conditions, hippocampal microglia isolated from VPA animals showed similar size and complexity (Fig. 6e) and shape profile (Fig. 6f–l) when compared with microglia isolated from control animals. In this case, internal complexity, circularity and cellular area are parameters of microglia reactivity [59, 62, 71, 83] (Fig. 6f). We then evaluated microglia reactivity when exposed to different stimuli. In response to LPS, hippocampal microglia both from control and VPA animals increased circularity (Fig. 6g–i). When exposed to synaptic terminals, hippocampal microglia isolated from both VPA and control animals responded by decreasing their cellular area (Fig. 6k, l). These results indicate that hippocampal microglia isolated from neonatal VPA animals and cultured in the absence of neurons showed preserved basal and stimuli-mediated reactivity. Since microglia reactivity profile is associated to cell functionality [82], our results suggest that hippocampal microglia from VPA rats are not intrinsically compromised.

## Discussion

This study provides in vivo and in vitro evidence that prenatal administration of VPA primes the neuronal population of the hippocampus to develop an altered synaptic pattern during early life neurodevelopment. We employed the VPA rat model since it mimics the behavioral, anatomical, synaptic and glial patterns described in ASD [40] and studied synaptic and microglia profiles in the hippocampus, a brain area associated to ASD impairments [44, 48, 49, 87]. We show in VPA animals that at early postnatal stages, before the behavioral deficits are evidenced (PND3), hippocampal synapse number and NCAM/PSA-NCAM balance are preserved and there is no sign of microgliosis in the CA3 region. On the contrary, concomitantly with the establishment of VPA behavioral deficits, in the juvenile period hippocampal synapse number is reduced and the NCAM/PSA-NCAM balance is increased in the absence of microgliosis. Thus, synapse number reduction is established postnatally after the neonatal period in the CA3 hippocampal region of VPA animals, most likely during the active periods of synaptogenesis and pruning [51, 74–76], and that synapse reduction is highly associated to behavioral deficits of VPA animals.

Importantly, our findings indicate that the synapse pattern seen in vivo in the hippocampus of VPA rats can be reproduced in vitro by neurons grown in the absence of glia. We cultured primary hippocampal neurons obtained from control or VPA pups and evaluated synapse pattern and structural plasticity in the absence of glia. As observed in vivo in VPA animals, their hippocampal neurons in vitro form a preserved number of synapses at the beginning of the synaptogenic period (DIV7) and develop fewer excitatory synapses after active synaptogenesis (DIV14). Similarly, hippocampal neurons isolated from VPA animals mimic at DIV14 the increase in NCAM/PSA-NCAM balance found at the juvenile period. Our work is the first to show that in the absence of glia, hippocampal neurons isolated from VPA animals exhibit the same synaptic pattern shown in vivo, suggesting that these cells are primed during the prenatal period. Moreover, this neuronal priming is enough to endorse the in vivo synaptic pattern. A recent work by Marchetto et al*.* [88] supports our findings by showing intrinsic alterations in induced pluripotent stem cell (iPSC)-derived neurons obtained from ASD patients. Moreover, we also show in a functional challenge that hippocampal neurons isolated from VPA animals exhibit a more resistant profile to glutamate-induced structural remodeling. Therefore, our work shows that hippocampal neurons isolated from VPA animals are more resistant to structural plasticity and suggests that the NCAM/PSA-NCAM balance could underlie such remodeling profile.

Another interesting finding is that hippocampal microglia do not seem affected at early stages of development or even when hippocampal synapse number is clearly reduced at the juvenile period (PND35). When comparing hippocampal microglia status described in the literature employing the VPA model, results are controversial and clearly dependent on microglia markers, hippocampal subregion studied and age of the animals [38, 39, 89, 90]. For instance, microgliosis has been described in the CA3 hippocampal region of VPA animals at PND35 by using tomato lectin [38] but no changes were observed when measuring Iba1 (+) cell number [39]. Besides, evidence of microgliosis is more consistently found in the CA1 hippocampal subfield and in older VPA animals [39, 89–91]. In the present study, we evaluated Iba1 (+) area, cell number and the proportion of ramified and unramified microglial cells in the CA3 hippocampal region of VPA animals at PND35. Assessment of microglia profile at this time point allows minimizing microglial changes triggered as adaptive or compensatory mechanisms. In our hands, hippocampal microglia of VPA animals show the same profile seen in control animals, either with normal or reduced number of synapses. To further study hippocampal microglia from VPA animals before the establishment of the reduction in synapse number, we isolated hippocampal microglia from control and VPA animals at PND3. Hippocampal microglia from VPA animals show the same basal and stimuli-mediated reactivity profile seen in microglia isolated from control animals. The fact that hippocampal microglia were purified at a time point when synapse number reduction was not present and that cells were differentiated in the absence of neurons allows us to propose that hippocampal microglia from VPA animals are not intrinsically altered.

In spite of the well-known hippocampal role in exploration and social interaction [43, 44], local connectivity and synaptic characteristics in this region remain elusive in ASD. Four well established hippocampal areas define the hippocampal circuit [77]. Several studies have addressed the excitability and morphology of the CA1 area in VPA-exposed animals. Particularly, Hajisoltani et al*.* [92] showed that pyramidal neurons from the CA1 of VPA animals depicted an altered excitability. In accordance with this finding, Fueta et al*.* [93] demonstrated alterations in CA1 excitability during the synaptogenic period which could correlate with the developmental delay observed in VPA animals. What is more, dendritic atrophy [94] and a smaller soma size [92] have been reported as morphological features of pyramidal neurons of the CA1 of VPA animals. The CA1 area represents the main output from the hippocampus circuit and receives intrahippocampal and distant area afferents. In opposition, even when the CA3 receives inputs from the entorhinal cortex onto the *stratum lacunosum-moleculare*, this area is mainly involved in intrahippocampal circuits: granular cells from the dentate gyrus innervate the apical dendrites of CA3 pyramidal neurons whose axons send collaterals back to the CA3 and to the apical dendrites of CA1 pyramidal neurons [77]. Thus, in our study, the CA3 synapse number reduction seen in the *stratum radiatum* of juvenile VPA animals suggests local circuit alterations in the hippocampus of these animals. Moreover, the evidence from hippocampal neurons in culture supports the in vivo findings and indicates that intrahippocampal connectivity is intrinsically affected in this experimental model of ASD. Interestingly, synaptic changes were absent in neonatal VPA animals, consistent with a preserved synapse number in cultured hippocampal neurons at the beginning of synaptogenesis in vitro. Cloarec et al*.* [95] have recently described an increase in hippocampal volume and longer apical dendrites of CA3 pyramidal neurons just after birth (PND0) in VPA rats, which highlights the importance of addressing different stages of hippocampal development and maturation.

Regarding the type of synapse, our work shows fewer excitatory synapses in the *stratum radiatum* of the CA3 hippocampal region of juvenile VPA rats. Consistently, isolated hippocampal neurons develop fewer glutamatergic synapses in vitro. Besides, functional labeling of presynaptic terminals shows that these neurons form fewer functional synapses containing smaller vesicular pools with conserved unloading kinetics. The reduction in vGLUT1 puncta size also supports the reduced size of pre-synaptic vesicular pools in neurons from VPA rats. In agreement with these results, puncta number and size of the constitutive glutamatergic NMDA receptor subunit NR1 were decreased in hippocampal neurons from VPA animals. A similar NR1 expression decrease has been found in the VPA rat hippocampus [96]. Thus, hippocampal neurons isolated from VPA animals exhibit an inherent capacity to form fewer excitatory synapses. Further investigation of the global effect on neuronal activity of all these synaptic changes will shed light on their impact on neuronal circuitry. It could be postulated that these synaptic features may contribute to a distinctive functional profile that would influence the behavioral phenotype in VPA animals. In fact, it has been shown that deletion of hippocampal NR1 leads to social impairment [97]. Moreover, the fact that social impairment is associated with NR1 decrease in the CA3 but not CA1 [97] highlights the role of intrahippocampal circuits in the social deficits of VPA rats.

Both glutamatergic and GABAergic neurotransmission have been proposed to be compromised in ASD patients. Regarding the former, mutations in genes which encode subunits of glutamatergic receptors [98, 99], alterations in the signaling pathway of metabotropic glutamate receptors [100] and increased blood glutamate levels [101] were reported in ASD patients. As to inhibitory neurotransmission, post-mortem samples from patients with ASD show a decrease in GABAa and GABAb receptors in the brain cortex and cerebellum [102–105]. However, a recent meta-analysis excludes the association between polymorphisms in GABA receptors, particularly linked to 15q11-q13 duplication, and the risk for ASD [106]. In the present work, we show that hippocampal neurons isolated from VPA animals form in vitro fewer glutamatergic pre- and post-synapses (vGLUT1 and PSD-95 puncta number, respectively) while GABAergic terminals (GAD-67 puncta) remain unaffected. These results emphasize the involvement of the glutamatergic neurotransmission in the altered hippocampal synaptic features in the VPA model, which has been reported deeply affected in the somatosensory cortex [107]. Related to this notion, levels of glutamate in the hippocampal-amygdala complex were found to be augmented in adult ASD patients [108] but conserved with a trend to reduction in adolescents [109], showing age heterogeneity. Moreover, the VPA model proved to mirror the glutamate reduction in the striatum observed in ASD patients with no changes in GABA levels, strengthening the face validity of this model and the implication of glutamate neurotransmission [110]. Contrary to the few studies addressing the glutamate system in the hippocampus, GABAergic neurotransmission has been much more described. A reduction in GAD-67 expression was reported in the whole hippocampus [73, 89] while a conserved number of parvalbumin-positive inhibitory interneurons was found in the CA1 of adult VPA animals [111]. Based on findings in humans, it is of great importance to address the interneuron population in each hippocampal subfield [112, 113].

Our study shows that hippocampal neurons in vitro also mimic other aspects described in ASD patients or experimental models. For instance, dendritic branching was found to be reduced in the hippocampus of ASD patients [49] and also in VPA animals [50, 94]. In our hands, cultured hippocampal neurons isolated from VPA animals exhibit a smaller dendritic arbor after active synaptogenesis in vitro. It should be mentioned that synapse formation is highly affected by neurite arborization [57, 78]. However, in hippocampal neurons isolated from VPA animals, synapse number diminishes regardless of dendritic shortening. Therefore, changes in the synaptogenic process seem to be involved in the decline of puncta number. In fact, synapse number reduction is established during the active period of synapse formation in vitro*,* strengthening the former idea. Another interestingly aspect is the NCAM/PSA-NCAM balance. Hippocampal neurons from VPA animals mimicked in vitro the increased NCAM/PSA-NCAM ratio seen in vivo. Since NCAM has been highly associated with glutamatergic synapses [114] and the number of excitatory synapses is lower, higher neuronal NCAM/PSA-NCAM ratio could suggest highly adhesive synapses [23, 115]. Accordingly, hippocampal neurons from VPA animals exposed to glutamate revealed a more resistant profile to structural synaptic remodeling. Our findings are in agreement with the decreased LTP described in the hippocampus of VPA animals [96]. Intriguingly, PSA removal from NCAM is known to increase NMDA receptor activity [21]. Also, diminution of PSA-NCAM levels precedes glutamate-induced synapse remodeling [57]. Thus, not only higher NCAM expression but also lower PSA-NCAM levels may contribute to synapse remodeling resistance in hippocampal neurons from VPA animals. Therefore, the distinctive functional response of hippocampal neurons isolated from VPA animals to glutamate-induced synapse remodeling uncovers their distinctive synaptic pattern in which NCAM/PSA-NCAM increase can be postulated to play a key role.

**Fig. 7:**
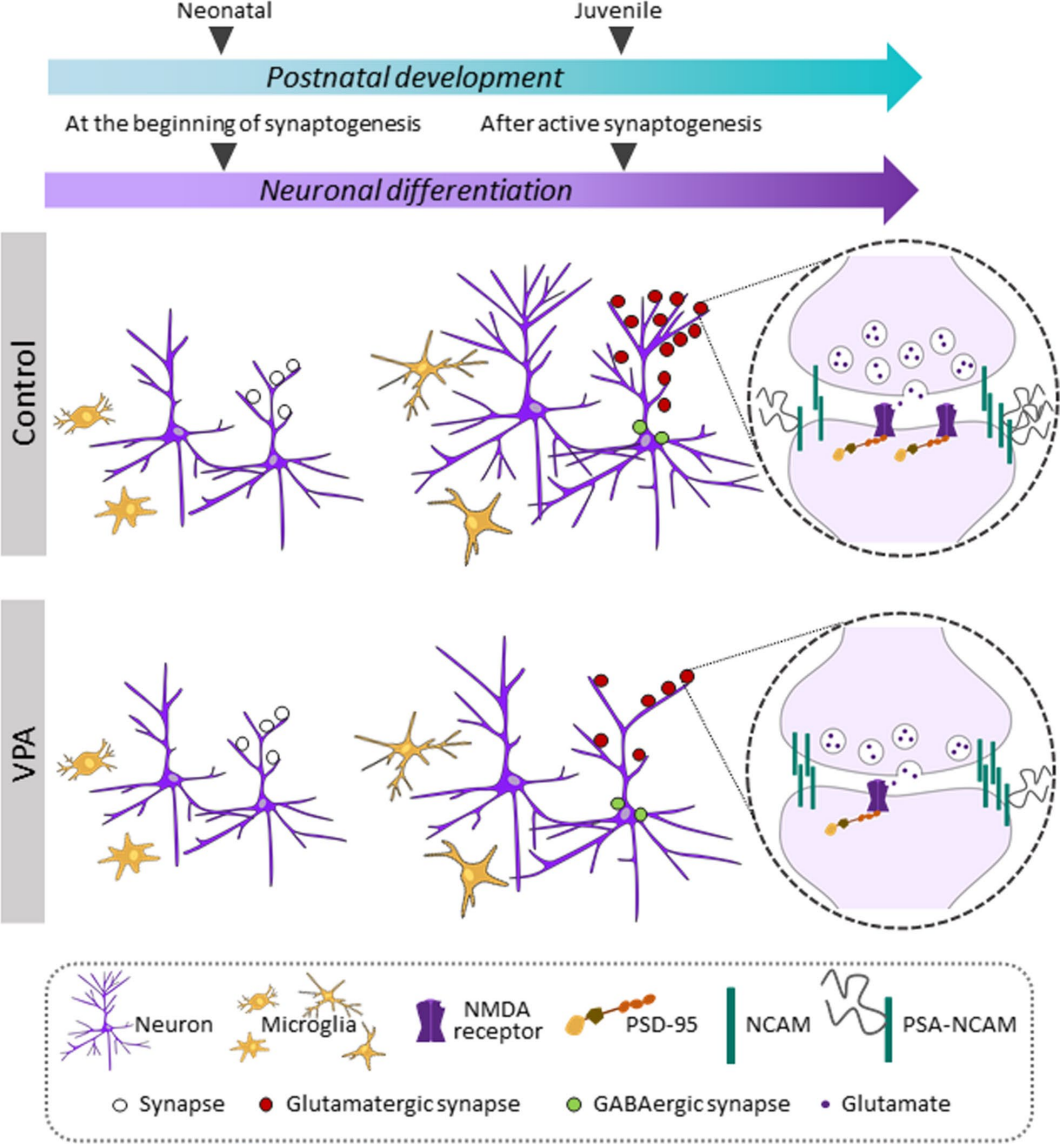
Schematic representation of time-dependent hippocampal alterations observed in the VPA model. The temporal trajectory of in vivo hippocampal synaptic alterations is mimicked in vitro by neurons cultured in the absence of glia. Hippocampal alterations in VPA animals develop after the neonatal period in vivo whereas the aberrant synaptic pattern of hippocampal neurons from VPA animals occurs after active synaptogenesis in vitro. Initially, neurons from VPA animals show preserved dendritic tree and synapse number according with developmental stage, but as differentiation proceeds they exhibit a smaller dendritic arbor and fewer glutamatergic synapses. In fact, the latter depicts distinctive features: smaller vesicular pool, fewer NMDA clusters, increased NCAM and reduced PSA-NCAM, which could contribute to a higher resistance to structural synaptic remodeling. On the other hand, hippocampal microglia proved to be unaltered in neonatal and juvenile VPA animals or in culture, suggesting it may not be primarily affected in the VPA model

To sum up, our findings are the first to show that hippocampal neurons from VPA animals possess an inherent capability to form fewer excitatory synapses and exhibit resistance to structural remodeling. In vitro*,* hippocampal neurons isolated from VPA animals mimic the in vivo synaptic pattern whereas hippocampal microglia isolated from VPA animals do not seem to be primarily affected (Fig. 7). Thus, we provide evidence that hippocampal neurons from VPA animals are intrinsically primed to acquire a distinctive synaptic pattern leading to a scenario where synapse formation and/or stability could be compromised. Our study highlights the crucial role of hippocampal neurons in the establishment of synaptic alterations in this brain region.

## Limitations

Although evidence emphasizes the existence of sex-dependent differences in behavior and neurobiology in ASD patients as well as in the VPA model, in this study, only male pups were chosen to perform the experiments. Therefore, conclusions driven from our results may or may not reflect behavioral and/or synaptic alterations in the female hippocampus. We worked only with male animals since we previously characterized behavior and hippocampal alterations in juvenile male VPA rats. Moreover, ASD is more often diagnosed in boys than girls and the behavioral and neurobiological features are better documented in male than in female VPA animals. Another issue to be considered is the excitatory/inhibitory balance in the hippocampus. GABAergic neurotransmission has been much more addressed than the glutamatergic system in patients and experimental models of ASD. Our study provides in vitro and in vivo evidence that excitatory synapses are diminished in the hippocampus of VPA animals. Although our in vitro findings suggest preserved GABAergic synapse number in hippocampal neurons in culture, further evaluation of synaptic terminals and interneurons in hippocampal subregions of VPA animals would shed light on excitatory/inhibitory balance in intrahippocampal circuits. Similarly, electrophysiological experiments could also contribute on this matter.

## Conclusions

Our study indicates that hippocampal neurons from VPA animals possess an inherent capability to form fewer excitatory synapses and exhibit resistance to structural plasticity most likely due to an increase in NCAM/PSA-NCAM balance. Hippocampal synapse number reduction is established postnatally after the neonatal period in the CA3 hippocampal region of VPA animals, possibly during the active periods of synaptogenesis and pruning and in close association with VPA behavioral deficits. Our study provides evidence that hippocampal neurons from VPA animals are intrinsically primed to acquire this atypical pattern whereas hippocampal microglia do not seem to be primarily affected. In this context, it could be suggested that microglia cells are not determinant for developing or counteracting the synaptic outcome in this brain region. Therefore, our study highlights the crucial role of hippocampal neurons and their structural plasticity in the establishment of the synaptic alterations observed in the VPA model.

## Data Availability

The datasets used and analyzed during the current study are available from the corresponding author on reasonable request.

## Abbreviations

ASD: Autism spectrum disorders
CA: Cornu Ammonis
CNQX: 6-Cyano-7-nitroquinoxaline-2,3-dione
DAPI: 4′,6-Diamidino-2-phenylindoledihydrochloride
DIV: Days in vitro
E: Embryonic day
GABA: Gamma-aminobutyric acid
GAD-67: Glutamic acid decarboxylase 67
Iba1: Ionized calcium-binding adapter molecule 1
IF: Immunofluorescence
iPSC: Induced pluripotent stem cell
LPS: Lipopolysaccharide
MAP-2: Microtubule-associated protein 2
MK-801: Dizocilpine (5S,10R-(+)-5-Methyl-10,11-dihydro-5H-dibenzo[a,d]cyclohepten-5,10-imine maleate
NCAM: Neural cell adhesion molecule
NMDA: *N*-Methyl-d-aspartate
NR1: NMDA-NR1 receptor subunit
PND: Postnatal day
PSA-NCAM: Polysialylated-neural cell adhesion molecule
PSD-95: Postsynaptic density protein-95
pyr: Soma of CA3 pyramidal neurons
RT: Room temperature
SD: Standard deviation
ST: Synaptosomes or synaptic terminals
SYN: Synaptophysin
TEM: Transmission electron microscopy
vGLUT1: Vesicular glutamate transporter 1
VPA: Valproic acid
WB: Western blot
